# Transforming UNICEF’s approach to health system strengthening: what place can a blended learning course play?

**DOI:** 10.1186/s12960-021-00553-8

**Published:** 2021-02-06

**Authors:** Lucio Naccarella, Barbara McPake, Prarthna Dayal, Waithira Gikonyo, Claudia Vivas Torrealba, Alison Morgan

**Affiliations:** 1 School of Population and Global Health, Centre for Health Policy, The University of Melbourne, Level 4, 207 Bouverie St., Melbourne, VIC 3010 Australia; 2 School of Population and Global Health, Nossal Institute for Global Health, The University of Melbourne, Melbourne, Australia; 3 Division of Data, Research and Policy, UNICEF, NYHW, New York, NY USA; 4 Health Section, Program Division, UNICEF, NYHQ, New York, NY USA

**Keywords:** Child health, Evaluation, Health systems

## Abstract

**Background:**

The United Nations Children’s Fund (UNICEF) published their Health Systems Strengthening (HSS) approach to meet its strategic goals of ending preventable maternal, newborn and child deaths and promoting the health and development of all children and reducing inequities in health in 2016. UNICEF commissioned the University of Melbourne’s Nossal Institute for Global Health to develop and deliver a pilot blended HSS program, involving 60 hours of online learning and 2 weeks of face-to-face teaching over a 6-month period. To assess the extent to which the HSS program had built the first 83 UNICEF 2017 graduates’ capabilities to apply HSS actions by 2017, UNICEF funded an independent evaluator from the University of Melbourne.

**Methods:**

A mixed-methods assessment was conducted using: online surveys of graduates at: enrolment, completion, 6 months post-HSS program; nine focus groups with graduates at face-to-face workshops; and interviews with purposive samples of UNICEF graduates and graduate Senior Managers 12 months post-HSS program.

**Results:**

The HSS program content, structure and mode of delivery was positively received. Graduates reported increased confidence taking HSS actions and multiple changes in work practices (e.g., increased systems thinking and using of health system-based approaches). Graduates’ Senior Manager interviews revealed mixed impressions of graduates applying HSS actions, partly explained by the fit between the HSS program learnings and UNICEF’s workplace environment. Key contextual factors influencing graduates applying HSS actions included: workload; limited opportunities to apply HSS actions; limited HSS examples; and variable support to apply HSS actions. Graduate and Senior Manager suggestions to optimise applying HSS actions included: linking HSS program content with UNICEF priorities; increasing opportunities for graduates to apply HSS actions; increasing access to HSS support.

**Conclusions:**

The paper concludes by presenting HSS program and assessment suggestions from the 2017 UNICEF Pilot HSS program assessment and actions taken for the 2018 UNICEF staff cohorts by HSS program developers, funders and beneficiaries.

## Background

The West African Ebola epidemic in 2014 drew the world’s attention to the importance of health system strengthening [4, 8, 11]. Responding to this crisis, in 2016 UNICEF published its Health Systems Strengthening (HSS) approach to meet its strategic goals of ending preventable maternal, newborn and child deaths and promoting the health and development of all children and reducing inequities in health. UNICEF defines HSS as “actions that establish sustained improvements in the provision, utilization, quality and efficiency of services delivered through the health system and encourage the adoption of healthy behaviours and practices” ([15], p 5). UNICEF identified the need for developing internal capacity for HSS, empowering its staff in different sectors at headquarters, regional offices and country offices to engage with confidence and lead the HSS work and integrate health in relevant UNICEF-supported programmes to optimise the impact on UNICEF’ work on women and children. UNICEF’s HSS approach connects national and subnational levels, focusing particularly on subnational management capacity and community engagement based upon sound national policy, plans and financing [15].

In 2016 UNICEF commissioned the University of Melbourne’s Nossal Institute for Global Health to develop and deliver a pilot blended HSS program. The HSS program consisted of 12 modules delivered online, each taking between 4 and 6 hours to complete, and including a module on Human Resources for Health (Box 1). This was followed by 2 weeks of face-to-face teaching covering the final two modules. The objectives of the whole HSS program as shown in the theory of change (ToC) (Box 2) developed by University of Melbourne’s Nossal Institute for Global Health to inform the 2017 Pilot HSS Program were to:

- 1.Institutionalise a systems approach in UNICEF country programs.
- 2.Position UNICEF as a credible strategic partner in supporting governments’ efforts to improve health systems, particularly for the benefit of disadvantaged children and women.

The Theory of Change outlined the enabling factors and assumptions underpinning a well crafted training program. It assumed that the training program would lead to increased competencies on HSS amongst UNICEF staff enabling them to identify systems level bottlenecks and implement HSS strategies within their country programs, and to interact more meaningfully with governments and other development partners to strengthen health systems for better health outcomes for the most vulnerable women and children.

In total 102 participants enrolled and 83 completed all training components of the 2017 Pilot HSS program. During the roll out of the 2017 Pilot HSS program, in addition to the external independent assessment described in this paper, the University of Melbourne’s Nossal Institute for Global Health conducted its own process assessment through three online anonymous surveys, requesting feedback from all participants at the completion of module 6, and then at the end of each of the two face-to-face teaching weeks. Participants were requested to comment on the learning package, the time taken to complete each module and whether the material was appropriate in content and delivery style. Participants were very satisfied with the content and mix of content on the online modules, with 75% stating they spent over four hours per week on the course, and valued the face-to-face modules for both the content, particularly the final week which involved a full week simulation of applying the course content to a country’s five year health reform agenda. Participants also valued the chance to learn from peers across the globe, with the 83 graduates being drawn from over 60 different country contexts.

The purpose of the study was to assess the extent to which the HSS program had built the first 83 UNICEF 2017 graduates’ capabilities to apply HSS actions’.

Box 1: UNICEF HSS program module and objectives outline *Module 1: Introduction to health systems and health systems strengthening*

Objective 1: Look back at HSS history.

Objective 2: Current frameworks for Health Systems Strengthening.

Objective 3: National to community Health Systems Strengthening.

Objective 4: Engaging other sectors in Health Systems Strengthening.

Objective 5: Players and power in HSS.

*Module 2: National level health systems*

Objective 1: Health planning cycle.

Objective 2: Meaning of equity.

Objective 3: Addressing inequities.

Objective 4: HSS actions.

*Module 3: UNICEF HSS analytical framework*

Objective 1: Background and rationale.

Objective 2: The seven steps.

Objective 3: Tanahashi framework and bottlenecks.

Objective 4: Strengths and challenges.

*Module 4: Subnational health systems*

Objective 1: District health systems.

Objective 2: District health systems strengthening.

Objective 3: Strategy selection.

Objective 4: Strategy implementation.

Objective 5: Applying DHSS.

*Module 5: Community health systems*

Objective 1: Principles of community health systems.

Objective 2: Community health workers.

Objective 3: Community level governance and accountability mechanisms.

Objective 4: Bringing it together: Intervening across the system.

*Module 6: Financing health systems*

Objective 1: Principles of UHC.

Objective 2: Health financing functions.

Objective 3: Prepayment and health insurance.

Objective 4: Government and private funding.

Objective 5: Fiscal space analysis.

Objective 6: Equity and efficiency.

Objective 7: National health accounts.

*Module 7: Human resources within health systems*

Objective 1: Health workforce: who are they and where are they?

Objective 2: Cadres, roles and task shifting.

Objective 3: Incentives and motivation.

Objective 4: Health workforce management and regulation.

*Module 8: Data for health systems strengthening*

Objective 1: Data for decision-making in HSS.

Objective 2: National and subnational data sets—strengths and weaknesses.

Objective 3: Major sources of data for HSS.

Objective 4: New technologies for data collection.

Objective 5: Challenges in interpreting data.

*Module 9: Supply chain management*

Objective 1: Supply chain management and the health system.

Objective 2: Supply chain management systems.

Objective 3: Developing resilient supply chain management systems.

*Module 10: Social protection within the health system*

Objective 1: Choice and deprivation.

Objective 2: Concept and models of social protection.

Objective 3: Relationship between child poverty and health.

Objective 4: Barriers.

Objective 5: Interventions to address social protection.

*Module 11: Quality of care in health systems*

Objective 1: Overview of Quality of care frameworks.

Objective 2: Examining the WHO quality of care framework.

Objective 3: Quality of care and Universal Health Coverage.

Objective 4: The importance of other sectors in quality care.

Objective 5: Monitoring quality of care.

Objective 6: System strategies for improving quality.

*Module 12: Private sector and civil society roles in health systems*

Objective 1: The private sector: characteristics and roles.

Objective 2: Private sector contributions.

Objective 3: Regulate and engage.

*Face-to-face teaching weeks*

*Module 13: The health system toolbox*

*Module 14: Systems thinking for HSS*

Box 2: 2017 Pilot HSS program theory of change

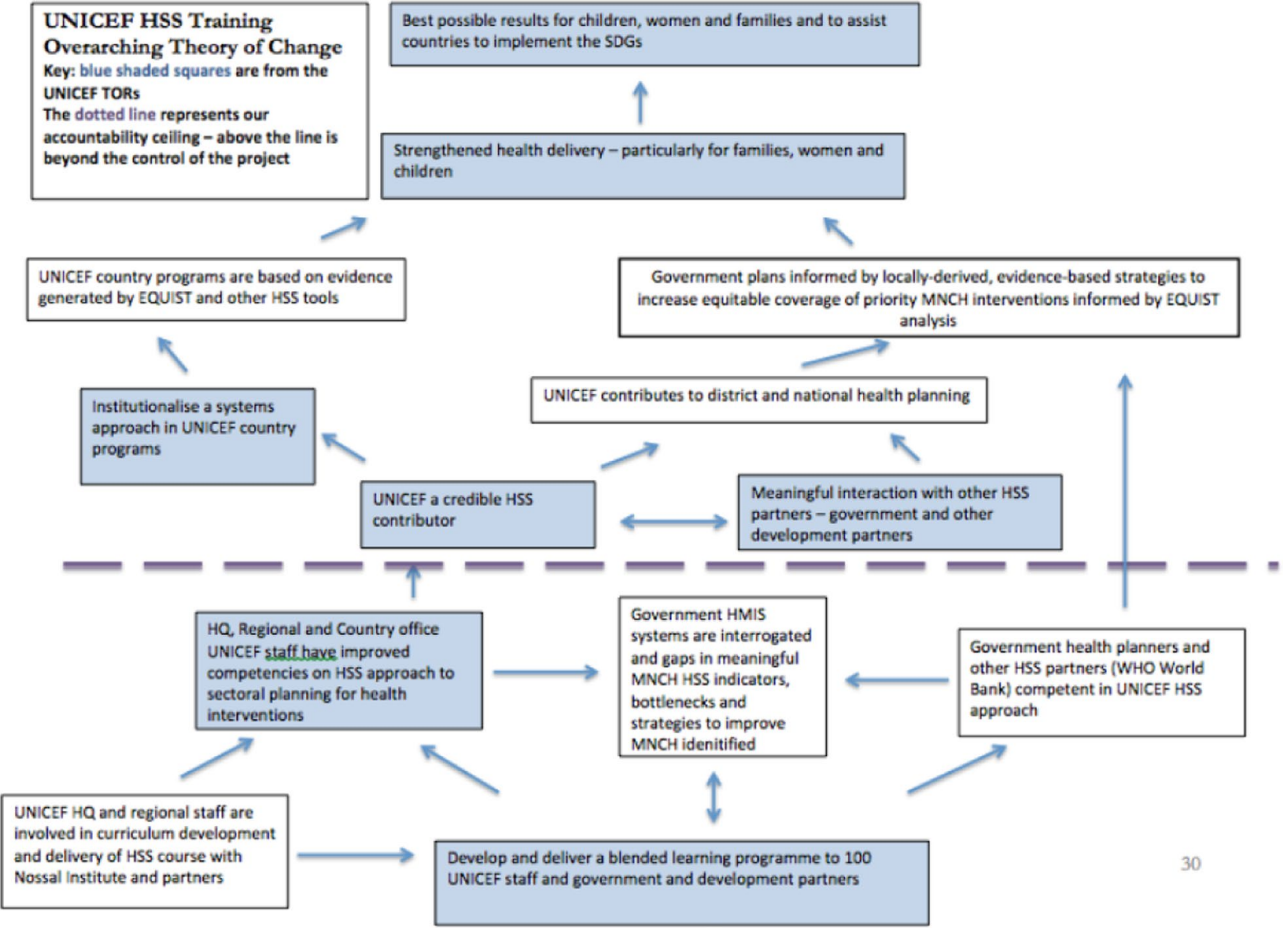

## Methods

To assess the extent to which the HSS program had built the first 83 UNICEF graduates’ capabilities to apply HSS actions or competencies (i.e. examples of where having an understanding of HSS will inform these actions), UNICEF funded an independent evaluator from the University of Melbourne (Box 3).

### HSS training program

The 12 online modules were designed so as to provide participants with the technical knowledge and the latest evidence on existing overall health systems strengthening frameworks and approaches, the different health system components and actions and introduce participants to a systems thinking approach to HSS (See Box 1 for further details on modules and their content). This knowledge is then developed by participants during the face-to-face training component where participants apply a systems thinking approach to analyse and solve a range of health system challenges.

The first week of the face-to-face training builds on materials from the online modules to further participants’ skills and critical thinking to address the wicked common problems plaguing health systems globally—not enough money, insufficient numbers and distribution of human resources, and weak measurement and accountability leading to poor quality services and inequity in health outcomes. Sessions include participatory activities aimed at equipping participants with competence in robust health systems planning, analysis and measuring progress, while drawing on their experiences to specifically address how participants can apply these skills to their day-to-day work with UNICEF.

The second week is the capstone module which draws on the learning of the entire course in confronting participants with health systems problems over five days. Modelled on the Harvard Business School Case Method, participants work in teams who are advisors to the Ministry of Health in a simulated country. Each day teams are asked to develop strategies and policies to address complex scenarios. Teams then debate approaches with each other and with facilitators. Participants are guided to grapple with the dynamic complexities of a detailed simulated health system, through examining real-world case studies in a variety of national and subnational health systems to address the problems of the day.

A hypothesised 2017 HSS Training Program logic model (Box 4) was developed by the independent evaluator from the University of Melbourne to provide a visual representation of the assumptions underpinning the Pilot (from the evaluators perspective) and the linkages between intended outcomes, activities, inputs and contextual factors.

Box 3: Examples of HSS competencies

1. Discuss health financing, national health accounts or fiscal space with Ministry of Health or Ministry of Finance staff
2. Develop strategies to address health workforce challenges in your country
3. Critique programmes designed to provide social protection
4. Analyse the supply chain implications of UNICEF programs delivered at the community level
5. Explain Universal Health Coverage to colleagues in non-health sectors
6. Design a quality improvement (QI) plan for maternal or newborn health services

Box 4: 2017 Pilot HSS program assessment logic model

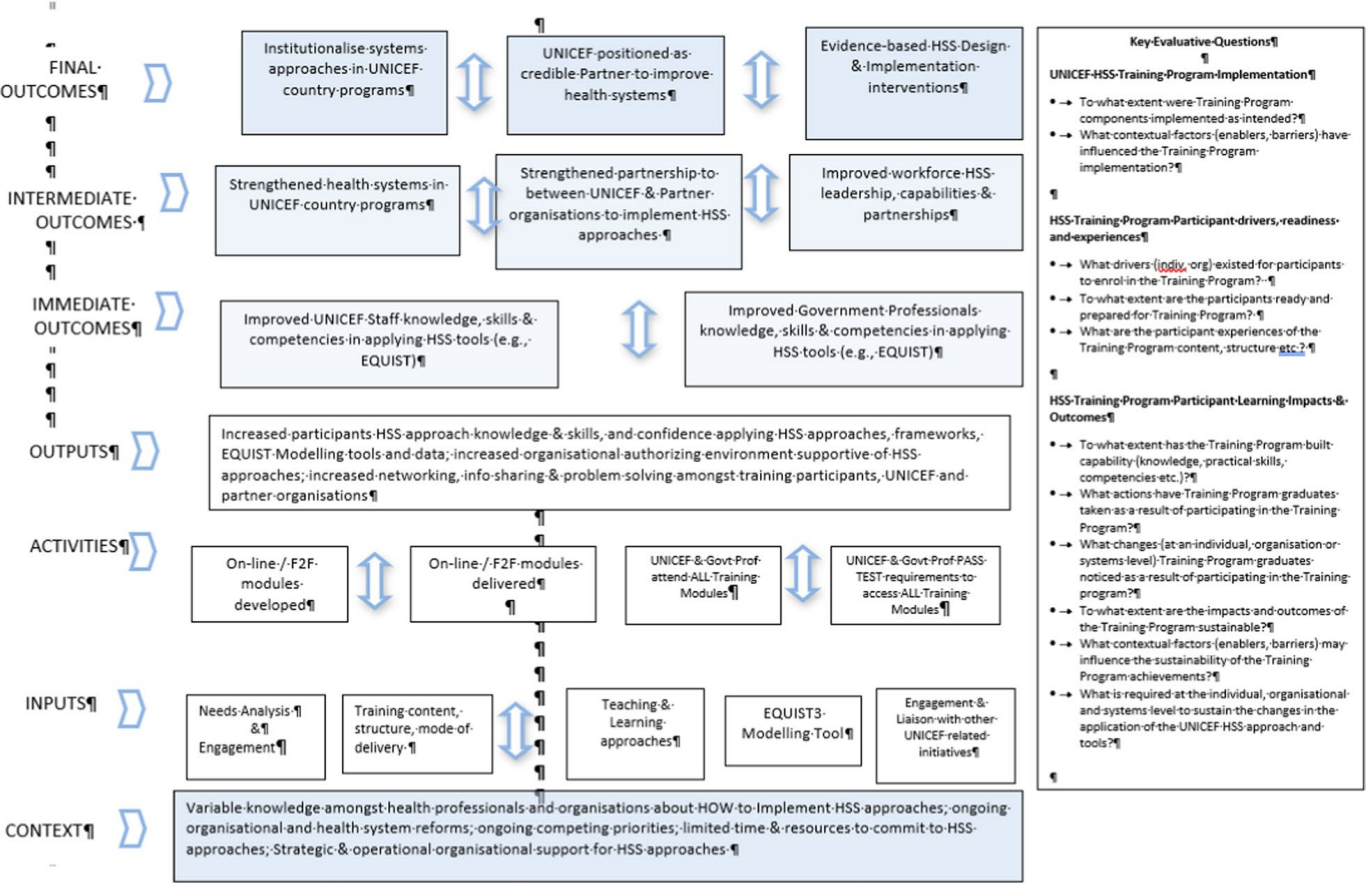

## Results

This section summarises the assessment findings under five headings, with illustrative quotes:

1. Assessment Study Participants
2. UNICEF graduates’ perceptions of HSS Program
3. Changes in UNICEF graduates’ confidence with applying HSS learning
4. HSS program impacts upon UNICEF graduates
5. Contextual factors influencing graduates’ capacity to applying HSS learning

### Assessment study participants

A total of 102 UNICEF staff enrolled in the 2017 HSS program*,* of which 83 completed all 14 HSS program modules, which is common in such programs. As can be seen from Box 5, overall there were differences in the number of UNICEF graduate respondents across the assessment activities (online surveys, interviews, focus groups) and in their UNICEF regions workplaces and work areas. For instance, UNICEF graduates reported working in multiple areas including: Health; Immunisation; Nutrition; Supply Chain Management; WASH; in differing roles; and working at country field offices, national offices, regional and headquarters. The study participants cannot be considered representative of the 83 graduates, nor are the three samples directly comparable. However, given the diversity of respondents across UNICEF country regions, work areas and roles, the assessment respondents are illustrative of HSS program graduates.

Table 1 provides a summary of the assessment methods, purposes, recruitment, and timing. The assessment built upon traditional approaches to evaluating professional development courses, focusing on: participant learning outcomes, intentions and confidence to use their newly acquired knowledge and skills; participant use of knowledge and skills gained; and participant perception of organisational support required to implement participant learning outcomes [6, 9].

**Table 1:** Summary of assessment methods, purpose, recruitment and timing

Methods and mode	Purpose	Recruitment	Timing
Online survey	To explore prior knowledge about HSS actions; perceptions of their readiness and their organisation to implement HSS approaches	All 2017 UNICEF enrolees invited via email to complete online survey	At HSS program enrolment
Online surveys	To explore experiences and perceptions of learning content, structure, mode of delivery; potential future actions regarding HSS actions	All 2017 UNICEF enrolees invited via email to complete online survey	At 6 weeks and completion of online HSS program
Focus group discussions—in person	To explore graduates’ experiences and perceptions of the HSS program	All 2017 UNICEF graduates Australia were invited to participate	At face-to-face modules 13 & 14 in Melbourne, Australia
Online survey	To explore experiences of implementing and applying HSS actions; organisational readiness and support required to implement graduates’ learnings	All 2017 UNICEF graduates invited via email to complete online survey	Six months post-HSS program
Individual semi-structured interviews—telephone or Skype	To explore to what extent learning changed: ways of thinking, work practices; and whether graduates were supported to apply learnings from HSS program	15 UNICEF graduates purposively sampled: across 7 UNICEF regions and roles were invited via email to participate	12 months post-HSS program
Individual semi-structured interviews—telephone or Skype	To understand and explore impacts and benefits of the HSS learning upon graduates, and work environment within which graduates return to after training	15 UNICEF Senior Managers of graduates purposively sampled: across 7 UNICEF regions and roles were invited via email to participate	12 months post-HSS program

To increase rigour, the assessment used multiple methods, and a transparent analysis and interpretation process. To enhance the utilisation of the assessment findings by HSS program funder and developers, participatory [2] and realist evaluation [10] evaluation approaches were used.

The online survey quantitative data were analysed using descriptive (frequencies) statistics. The online surveys (free text responses) were analysed using the constant comparative thematic analysis approach [12]. The assessment questions guided the quantitative and qualitative analyses. The assessment received ethics approval [Ethics ID: 1647861.1] from The University of Melbourne Human Ethics Advisory Group.

Box 5: Assessment participants A total of 102 UNICEF staff participated in the 2017 HSS Program. Of these:

- 53 of 102 (52%) UNICEF participants completed an online survey at HSS program enrolment
- 79 of 83 (95%) UNICEF graduates completed survey at HSS program completion
- 73 of 83 (88%) UNICEF graduates participated in focus group discussions at the HSS program face-to-face workshops
- 35 of 83 (42%) UNICEF graduates completed an online survey 6 months post-HSS program
- 10 (out of 83) UNICEF graduates participated in a semi-structured telephone/Skype interview 12 months post-HSS program
- 7 UNICEF graduates’ Senior Managers participated in a semi-structured telephone/Skype interview 12 months post-HSS program

## UNICEF graduates perceptions of HSS program

### Program content

Overall graduates reported that the HSS program content was good (“content of the program was good and thought provoking”), comprehensive (“covered all basic components of health system very well in a very comprehensive manner”) and aligned with key frameworks and strategies and (“in line with World Health Organization (WHO) Building Blocks and UNICEF’s Health strategy”).

Overall the participants found the course content thought provoking, comprehensive and aligned with key health system frameworks. Suggestions for improvements included diversifying the context of country case studies, reducing the time spent learning how to use a range of computer programs used in health system strengthening and putting more focus on resilient health systems in complex emergency settings.

### Program structure

Overall UNICEF graduates responded positively to the HSS Program structure as demonstrated by the following quotes:

“well structured starting with framework and general concepts and then deep diving into specific components of HSS”; and Very good- the structure allowed for all elements to be covered with minimal wastage)

Graduates also commented that there was a good mix of theories with practice.

UNICEF graduates offered multiple suggestions about improving the HSS program structure, including having more specific objectives and more online discussions. The face-to-face modules were perceived as having too many HSS tools and that more exposure to the HSS tools was required prior to the face-to face modules.

### Program delivery

Overall graduates commented that the program delivery had the right combination of visual and, audio, in class activity; pragmatic online modules; diverse methodology (“a good mix of lectures, role plays and group learning”) and great presenters (“all the presenters were very clear in their deliberations”). Graduates responded positively to the face-to-face modules, as they valued the opportunity to interact with HSS program deliverers and other UNICEF staff trainees (“Good balance between online and face-to-face course”; online phase with lecture materials very helpful. Face-to-face component with group sessions to tackle real-life scenarios—excellent).

UNICEF graduates offered multiple suggestions about improving the HSS program delivery, including: using university mentors to facilitate discussions during online course; to provide transcripts with videos; and reducing plenary presentations and increase floor for trainee experiences.

The UNICEF graduate interviews provided rich and broader insights into the HSS program content and context, particularly that the HSS program was more than the HSS tools as can be seen below:

“…the Learning [Program] was more than [HSS] tools- an opportunity to see the whole health system and understand how it works and what components and to move from an academic point of view to practice. Three key learnings. 1. understanding the health system- people, resources and data. 2. Interacting with different colleagues from different regions and different contexts and 3. using tools, but the weakest learning for me as no data. HSS tools are important but not the heart of the work—it’s the people, main issue is to understand that information is important, but need to deal with people. People are at the centre for community participation; transforming the situation and for meaningful relationships with Ministry of Health”.

The focus group discussions with UNICEF graduates also revealed the broader HSS program context. For example, one graduate commented on the significance of the HSS program*:*

“HSS is a game changer—signifies paradigm shift. UNICEF was a service delivery organisation, focussed on downstream, to now more upstream, analysing health system data”.

Collectively the assessment activities with the UNICEF graduates revealed multiple other issues relevant to the HSS program context that can be summarised into two categories:

- Role of UNICEF and purpose of the HSS program—several graduates raised questions at the strategic and operational level. For example: “What is the role of UNICEF in HSS?” and “What is the objective of UNICEF HSS training?”; “Is it to apply HSS Tools and Actions -as to apply HSS Tools need more technical, expert and consultancy support”
- Barriers for UNICEF adopting HSS approaches—graduates also raised multiple challenges for UNICEF adopting HSS approaches. For example: “Silos exist at a system, sector, organisation and community level”; “there is limited regional level quality and available data, mainly at country Level”; “there are competing priorities”; “donors drive priorities but there is variable understanding of HSS; and “the Ministry focuses on quick wins not systemic change”

### UNICEF graduates’ confidence with taking HSS actions

**Fig. 1:**
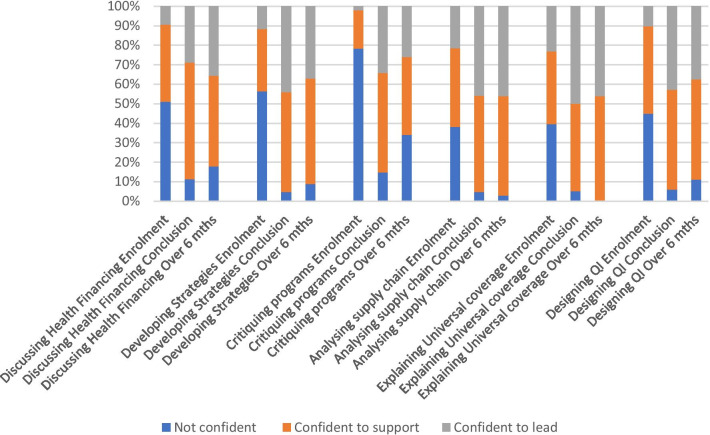
Changes in UNICEF graduates confidence with taking HSS actions

All UNICEF graduates were asked in the online surveys to rate their confidence with the examples of HSS competencies (Box 3) over three time points (1) at enrolment; (2) program conclusion and (3) 6 months post-program. As can be seen from Fig. 1:

- a greater proportion of UNICEF graduates reported being confident to support and to lead the HSS actions
- UNICEF graduates reported the most confident to support and lead ‘Explaining universal health coverage’; followed by ‘Analysing supply chain systems’
- over 6 months post-program a smaller proportion of UNICEF graduate reported being ‘not confident’ with all taking the HSS actions
- UNICEF graduates require further support with ‘Critiquing programmes designed to provide social protection’ and ‘Discussing health financing’.

### HSS program impacts upon UNICEF graduates

Evidence of the multiple HSS program impacts (i.e. observable changes in work practices) upon HSS graduates was evident from the online survey results at HSS program completion and 6 months post-HSS program*.* UNICEF graduates reported a spectrum of examples illustrating changes in work practices as a result of participating in the HSS program. Table 2 summarises the reported changes in work practices with illustrative quotes from both the online surveys (free text) and the semi-structured interviews—which all align.

**Table 2:** Changes in UNICEF graduates work practices

Reported changes in work practices	Online survey Illustrative quotes	Interviews Illustrative quotes
Changed thinking	Systems thinking is the major change that I have made in my daily work practice	HSS learning has helped me to think more systematically and holistically while planning… The seven steps of planning have been ingrained into my thinking
Increased knowledge, skills and confidence to apply HSS tools	Confident in contributing to GAVI HSS proposal writing and with appreciation of data analysis using EQUIST, it was easy to articulate Equity issues in the proposal	I became more explicit about HSS in my meetings with the MOPH. For example, when we proposed to host the management of Accelerated immunisation activities in the district offices (instead of the central) I used arguments in favour of decentralisation acquired in the course
Facilitated discussing HSS	A good understanding of HSS has enabled me to provide added value in the way we support the Government	I find myself advocating more and more for systems building approaches as opposed to disease specific activities. I apply the principles of the UNICEF HSS strategy and consider health systems at facility and community levels rather than vertical programmes. This has been made easier as more and more donors and partners prefer to support broader systems strengthening
Increased use of health system-based approaches	Focussing more on equity analysis and programming	I work in a very specialised area of work that tends to be very much a silo. Thinking about it now, I have actually moved to working in a more systemised approach…

A key quote illustrating the multi-level and multi-dimensional impacts upon UNICEF graduates work practices can be seen below:

“..there have been gradual shifts in my daily work, for example, relooking at the design of an on-going project on closing immunization equities in rapidly urbanizing settings in selected countries in East Asia, moving from specific programme focus to broader systematic approaches, increasing attention on the programmatic elements related to six health systems blocks, testing solutions in tackling the systematic bottlenecks associated with public financing and human resources constraints, more proactively engaging leaders of communities”.

The semi-structured interviews with the UNICEF graduates Senior Managers revealed mixed impressions about graduates applying HSS actions. Senior Managers confirmed that graduates had increased HSS awareness and knowledge and that the HSS program had broaden graduates worldviews, as can be seen below.

- HSS has really helped broaden [the graduates] and other graduates views and vision
- …learning has exposed [graduates] to the bigger picture and to be more affirmative…with HSS exposure they have more power and a different way to see the world- big picture lens
- A bigger outlook on the world
  However, only discrete (not widespread) and mixed examples of applying HSS actions were reported. Illustrative quotes from Senior Managers are provided below:
- increased awareness of HSS has led to HSS issues being put onto the table, I can see more coherent discussion of HSS, indicating increased confidence
- Refined planning in a systematic manner at both state and district level… facilitated assessment of initiatives, and reinforced need for data to support systemic structure and processes
- [HSS graduate] planned to do a nutrition conference—very technical, but returning from Melbourne they changed to planning a Nutrition Summit with key result areas, involving Ministry of Health, and a governance structure
- Graduate did the course, came back but never really applied what they learnt—but others who have done course have applied it- I can see it in their next workplans, using systems thinking, data—definitely it has made a difference

Several Senior Managers also commented that there was variability amongst UNICEF graduates who participated in HSS program in relation to evidence of putting HSS principles and concepts into graduates work practices—as can be seen below:

- I don’t have any real evidence—that HSS learning has influenced day-to-day work—more at a general discussion level
- HSS requires a big vision—but I have not seen it—no evidence that HSS has influenced their strategic thinking
- Yes I can see how graduates has used training in her monitoring and tracking country data using HSS concepts”

### Factors influencing UNICEF graduates applying HSS actions

The assessment activities revealed multiple contextual factors that were influencing UNICEF graduates applying HSS action that can be summarised into four key domains. Table 3 provides a summary of key contextual factors with illustrative quotes.

**Table 3:** Key contextual factors and illustrative quotes

Key contextual factors	Illustrative quotes
Graduates workload and work foci	In [country]—workload locally is huge (all about reacting to crises)—have no time to strategise and use HSS concepts in day-to-day work
Limited opportunity to apply HSS approaches	Course is designed very well, but staff struggle to make time to find opportunities to apply HSS approaches—need to look for opportunities in real world to apply HSS—the disconnect ‘it is not happening…I have tried to find opportunities for staff to apply HSS—as I am a strong advocate of HSS—but they [staff member] are resistant to data analysis systems, we have EQUIST tool—but they do not used it—they say—not everybody likes data—but it is public health
Limited HSS examples	Overall HSS course very useful—need more examples, need macro pictures to enable staff to think through other lens—a whole health sector lens
Varied supportive workplace environments	When trainees go back to work—management needs to see agenda in the same way—or else will just see HSS as a theoretical concept with no practical applications

UNICEF graduates and Senior Managers offered multiple suggestions to optimise UNICEF graduates applying HSS actions that can be summarised into four domains:

1. *HSS program content:* Revise program content to link with UNICEF priorities and programs
2. *HSS program structure and mode of delivery:* Refine structure and mode of delivery to increase opportunities for trainees to interact with program deliverers and other trainees
3. *Opportunities to apply HSS actions:* increasing opportunities for graduates to apply HSS actions upon return to workplaces
4. *Access to support:* Increased access to HSS technical and coaching support at a country level

A majority of UNICEF graduates Senior Managers interviewed also mentioned the need for a HSS Masterclass for Senior Managers, as seen below:

- Masterclass for UNICEF Senior Managers… as well succinct modules on HSS
- For Senior Executives—leaders like us—we can have a succinct HSS module for us
- Need a management level HSS Masterclass so that we can interact with colleagues who attended HSS training

Senior Managers also recommended more accountability for UNICEF graduates applying HSS can be seen below:

- HSS needs to be a key result area in the workplace—get it into the staff KPIs—need accountability.

## Discussion

This assessment has confirmed that the HSS program can be a vehicle for building UNICEF graduates capabilities to apply HSS actions to meet UNICEF’s strategic goals of ending preventable maternal, newborn and child deaths and promoting the health and development of all children and reducing inequities in health.

At an operational level, the HSS program has clear strengths including: program content, structure and mode of delivery that provides a mix of theory and practice, engaging with diverse visuals, audios and presenters.

While HSS program graduates reported increased confidence with HSS actions and changes in work practices, the HSS program faces key contextual challenges: graduate workload and work foci; limited opportunity to apply HSS approaches; limited HSS examples; and varied supportive workplace environments. These challenges require us to reflect upon the fit between the HSS program learnings and UNICEF’s enabling or not enabling workplace environment for its graduates to apply HSS actions. These findings are in line with the formative evaluation findings of UNICEF’s programming in HSS [7].

The following section contextualises and discusses the assessment findings with regard to two key domains: (1) Health Systems Strengthening policy context; (2) Transformative professional development transfer and effects and UNICEF’s Workplace Environment; and (3) the hypothesised Pilot HSS Program logic model. Reflections upon the assessment strengths and limtation are also discussed.

### Health systems strenghtening policy context

The assessment has revealed that the HSS program has contributed to increased UNICEF graduates’ HSS knowledge, skills, confidence and capabilities to contribute to HSS in their contexts. While these findings are indicators of the HSS program effectiveness, and provide evidence to suggest that a HSS paradigm shift is occuring across UNICEF and its graduates, it cannot be attributed solely to the HSS program. Over the 2017 HSS program period UNICEF initiated multiple other initiatives to advance HSS which also involved UNICEF gradutaes, thus potentially contributing to increased awareness and understandings about HSS approaches and actions. The assessment of the HSS program needs to view its graduates within an ecosystem framework—where the organisation and interaction of the HSS initiatives in the system are as important as the initiatives themselves.

### Transformative professional development transfer and effects and UNICEF’s work environment

The assessment findings revealed that graduates Senior Manager interviews had mixed impressions of graduates applying HSS actions upon their return to their workplaces. These findings require reflection for several reasons.

Firstly, professional development programmes are recognised as a means and not an endpoint and part of a transformative change process, involving a process of ongoing change, adoption, implementation, dissemination, and sustainability of the innovation into practice [3].

Secondly, based upon the reported challenges to applying HSS actions and the findings that UNICEF graduates requested more ongoing support, experiences and opportunities to applying HSS actions, it is important to reflect upon evidence about factors that influence learning transfer and effects.

Learning transfer is recognised as a complex phenomenon [13] which presupposes a series of stages and is based upon:

•Attendance—UNICEF graduates have attended the HSS program.
•Completion—UNICEF graduates having completed the HSS program.
•Knowledge recitation and retention—UNICEF graduates can recite and have retained HSS knowledge.
•Decision-making competence—UNICEF graduates know what to do with HSS actions.
•Task competence—UNICEF graduates use HSS knowledge.
•Transfer—UNICEF graduates use HSS actions; and
•Effects of transfer—training influences outcomes of UNICEF graduates work practices.

While a substantial literature exists [1, 5, 14] from multiple disciplines (management, training, professional development, adult learning and psychology) on learning program transfer and its effects, three key conceptual factors influence learning transfer: (1) graduate/individual characteristics; (2) training program design and delivery; and (3) work environment. These three learning and effects factors will now be briefly discussed in relation to the HSS program.

*UNICEF graduates/individual characteristics:* related to HSS program the following issues need reflection: graduates needs and goals related to HSS in their work; graduates competency to apply HSS; graduates motivations (intrinsic vs extrinsic) for applying HSS approaches; graduates career aspirations; graduates personality traits (e.g., openness to new experiences, conscientiousness), and graduates perceived value of HSS training.

*HSS learning program design-related to HSS program the following issues need reflection*: HSS training content, relevance and alignment to graduates work practices; HSS training presenter quality and engagement; provision of instructional strategies and methods to facilitate transfer of HSS approaches into practice (e.g., providing opportunities for adequate practice, feedback, reinforcement of applying HSS; opportunities for repeated practice of HSS approaches in “real” work practices at country level; active learning—involving trainees in course materials delivery; provision of self-management strategies to equip graduates to transfer HSS learning into practice.

*UNICEF workplace environment-related to HSS program the following issues need reflection:* the strategic link of the HSS training to UNICEF graduates work practices; the extent to which the learning transfer climate is supportive of HSS via management, supervisory and peer support and accountability system; extent of opportunities to use HSS knowledge and skills in work practices.

### Hypothesised pilot HSS program logic model

The hypothesised program logic (Box 4) underpinning the HSS program was largely confirmed. The hypothesised contextual factors were confirmed and expanded upon and remain key issues needing to be recognised including: UNICEF staff workload; limited opportunities to apply HSS actions; limited HSS examples; and variable support to apply HSS actions.

The hypothesised inputs and activities necessary for the HSS Training Program to work were also largely confirmed—given the positive comments on the program content, structure and mode of delivery.

The assessment has expanded the hypothesised HSS Program outputs, as UNICEF graduates reported that the HSS training had not only improved their knowledge, skills and confidence taking HSS actions and using HSS tools, but changed their thinking and work practices (e.g., increased systems thinking and using of health system-based approaches).

The short assessment time frame limits our ability to comment on intermediate and long-term Outcomes achieved so far. However, the assessment has revealed that the HSS Training Program is contributing to transformative changes (change in HSS mindsets and practices) at the individual and organisational level.

The scope and short assessment time frame also limits our ability to compare or comment on HSS Training Program with other innovative learning approaches’.

### Assessment strengths and limitations

The evaluator recognised that the HSS program developers (Nossal Institute for Global Health and UNICEF) and funded by UNICEF were the primary intended users of the assessment and hence required useful, specific and contextualised evidence to inform future HSS programs. A participatory and realist assessment approach guided the assessment methods, analyses and interpretations. The assessment findings reveal that the assessment approach facilitated a working partnership between the HSS program developers and the evaluator, leading to increased assessment utilisation and fostering a culture of learning between all.

We acknowledge that despite the assessment approaches, the assessment had limitations. The sample sizes in the assessment activities were small and not representative of the entire UNICEF HSS graduate cohort (*n* = 83) nor of UNICEF graduates who participated in other assessment activities by UNICEF and/or HSS program developers—whose perceptions of the HSS program were more positive. Despite inviting via email all graduates to complete online surveys, participate in interviews, the evaluator was unable to involve all trainees in the assessment, notwithstanding many invitations and email reminder attempts. We also recognise that from the outset the assessment had a particular focus upon UNICEF graduates applying HSS actions, while the learning program had a broader HSS focus.

We also recognise that being a pilot the HSS program has evolved significantly since 2017 and the assessment is therefore limited to the 2017 graduates not those who have completed the course in subsequent years.

## Conclusions

The assessment of the HSS program for the 2017 UNICEF graduates led to HSS program development and delivery and assessment suggestions for the HSS program developers (Nossal Institute for Global Health and UNICEF); and HSS program beneficiaries (UNICEF Headquarters, Regional and Country offices) many of which have been acted upon for the 2018 UNICEF staff cohort. Table 4 presents a summary of 2017 HSS program assessment suggestions and actions taken for the 2018 HSS program UNICEF Cohort.

**Table 4:** Summary of 2017 assessment HSS program suggestions and actions taken for 2018 UNICEF cohort

2017 Assessment HSS program suggestions	Actions taken for 2018 UNICEF staff cohort
*HSS Program Developers (Nossal Institute for Global Health and UNICEF)*	
Assess UNICEF staff HSS needs, goals and motivations pre-HSS program	Aligns with the formative evaluation findings of UNICEF’s programming in HSS [7] and has been acted upon
Increase strategic link of HSS actions to staff operational work level and practices	Strategic link of HSS actions to UNICEF staff operational work level and practices have been highlighted in the 2018 HSS program
Include diverse country case studies on how to apply HSS actions	
*UNICEF Headquarters*	
Invest in a UNICEF Senior Executive HSS Masterclass to facilitate them to support and authorise their graduates to apply HSS approaches	A Massive Open Online Course (MOOC) that was developed in 2018 and launched in 2019 provided a means for both UNICEF staff and development partners to become familiar with much of the course content. In addition, the health specialists in health systems at UNICEF headquarter runs regional webinars to follow up with HSS graduates and their senior staff
Include applying HSS actions’ indicators in UNICEF staff Performance Appraisal Frameworks: to optimise graduates accountability for applying HSS actions	UNICEF performance appraisals provide the opportunity, and in additional there is the potential to review graduate workplans for evidence of HSS application
Support UNICEF graduates exchange visits to countries with exemplar HSS actions	The suggestion of ‘staff exchange visits’ is met through the current regional HSS webinars conducted by the HSS team in headquarters where graduates chare their examples of actions taken with those from other settings
Provide more face-to-face opportunities for graduates to interact with program deliverers and other graduates post-course	In 2018 graduates of the HSS program were invited to facilitate a side event at the Global Symposium on Health Systems Research, the biennial conference for those involved in health systems research and practice
Invest in an expansion of the HSS program to UNICEF external partners	The launch of the MOOC in 2019 has been an excellent example of expanding the audience of the HSS program and reflects the regard with which UNICEF hold the blended learning program. In the first delivery of the MOOC over 6000 participants from 167 countries enrolled

A HSS MOOC has been developed by a collaboration between UNICEF and the Nossal Institute for Global Health and is available https://www.futurelearn.com/courses/health-systems-strengthening.

The 2017 assessment revealed the complex array of factors influencing UNICEF graduates applying their HSS learning, in particular, the UNICEF workplace environment. Given UNICEF’s commitment to adopting and applying HSS actions, the following research and assessment priority was suggested.

•To explore the influence of the work environment upon HSS program graduates applying HSS approaches.

At the time of the writing of this manuscript, UNICEF had commissioned a further assessment of the influence of the UNICEF work environment upon its 2018 HSS program graduates applying HSS actions, approaches and competencies in practice.

## Data Availability

Not applicable.

## Abbreviations

ECARO: Europe and Central Asia Regional Office
ESARO: Eastern and Southern Africa Regional Office
EAPRO: East Asia and the Pacific Regional Office
EQUIST: EQUitable Impact Sensitive Tool
F2F: Face to face
HQ: Headquarters
HSS: Health systems strengthening
LACRO: Latin America and Caribbean Regional Office
MENARO: Middle East and North Africa Regional Office
MNCH: Maternal Newborn and Child Health
MOOC: Massive open online course
QI: Quality improvement
SARO: South Asia Regional Office
ToC: Theory of change
TOR: Terms of reference
UNICEF: United Nations Children’s Fund
WASH: Water, sanitation and hygiene
WCARO: West and Central Africa Regional Office
WHO: World Health Organization
